# Trajectory Modulation for Impact Reducing of Lower-Limb Exoskeletons

**DOI:** 10.3390/mi13060816

**Published:** 2022-05-24

**Authors:** Long Zhang, Guangkui Song, Chaobin Zou, Rui Huang, Hong Cheng, Dekun Hu

**Affiliations:** 1 Center for Robotics, University of Electronic Science and Technology of China, No. 2006 Xiyuan Ave, Chengdu 611731, China; zhanglonguestc@163.com (L.Z.); sgk095@gmail.com (G.S.); chaubyzou@163.com (C.Z.); hcheng@uestc.edu.cn (H.C.); 2 Buffalo Robot Tech Co., Ltd., No. 888 Tianfu Ave, Chengdu 610213, China; hudekun@buffalo-robot.com

**Keywords:** exoskeletons, impact reducing, trajectory modulation

## Abstract

Lower-limb exoskeletons have received considerable attention because of their effectiveness in walking assistance and rehabilitation for paraplegic patients. Excessive foot–ground impacts during walking make patients uncomfortable and even lead to injury. In this paper, we propose an optimized knee trajectory modulation (OKTM) for foot–ground impact reduction. The OKTM can reduce the peak of ground reaction force (PGRF) by knee-joint trajectory modulation based on a parameters-optimizing spring-damping system. In addition, a hip trajectory modulation (HTM) is presented to compensate for torso pitch deflections due to the OKTM. Unlike traditional mechanical-device-based methods, the proposed OKTM and HTM require no bulky mechanical structures, and can adaptively adjust parameters to adapt to different impacts. We demonstrated the efficiency of the proposed approach in both simulations and experiments for engineering verifications. Results show that the approach can effectively reduce PGRF.

## 1. Introduction

Lower-limb exoskeletons are wearable devices that can detect human intentions and assist in walking, and have attracted significant interest in the past decades. Paraplegic patients can use lower-limb exoskeletons for walking in home scenarios or exoskeleton-based rehabilitation training in hospitals. A considerable number of studies has demonstrated their effectiveness [1,2,3].

**Figure 1:**
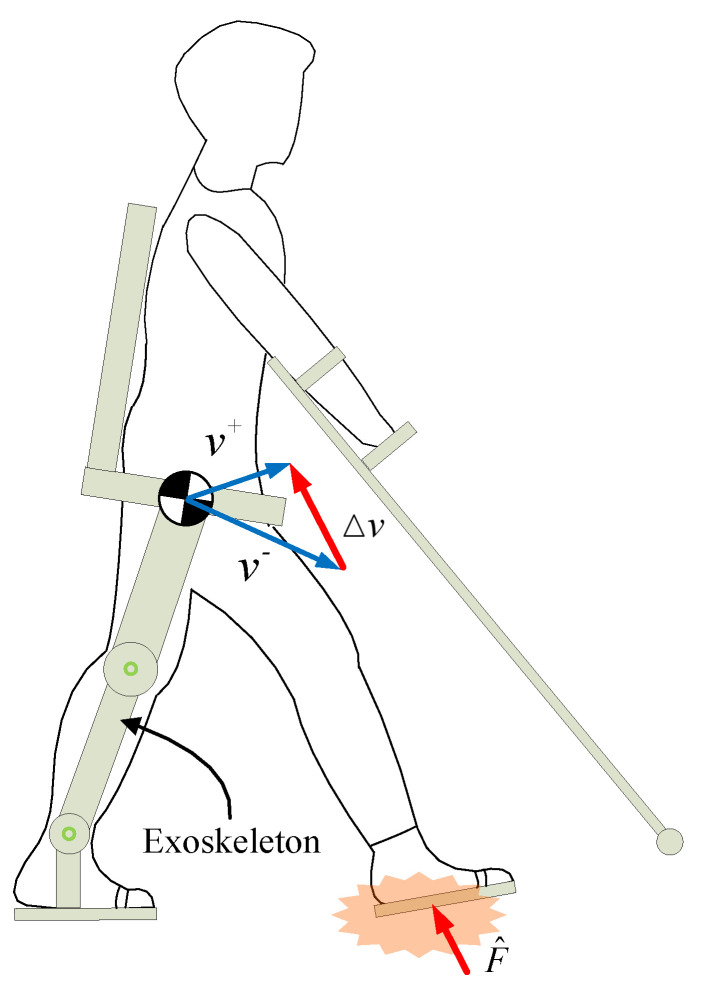
A subject is walking with a lower-limb exoskeleton. The CoM velocity is instantaneously turned from $v^{-}$ to $v^{+}$ when the swing foot strikes, and an impact force acts on the human-exoskeleton system. How to reduce the peak of ground reaction force?

Most lower-limb exoskeletons are fixed following predefined gaits collected from human clinical walking trials, such as ReWalk [4], EKSO [5], and Indego [6], etc. The center-of-mass (CoM) velocity of human-exoskeleton systems is redirected when swing feet hit the ground, and the speed variation Δv results in the impact force $\hat{F}$ acting on the foot plantar [7], as shown in Figure 1. The impact energy is gradually transmitted to the patient’s lower limb and trunk, to be ultimately absorbed by soft tissue, muscles, etc. [8]. The damage of the impact mainly comes from the peak of ground reaction force (PGRF). The excessive PGRF not only makes the patient uncomfortable, but may also leads to tissue damage and pain [7,8]. Therefore, the design of exoskeletons should be concerned with reducing the damage caused by the PGRF to patients.

Walking-impact reduction methods for biped walking can be divided into two categories. One is configuring shock-absorbing devices in lower limbs. Another category is changing the joints configuration of lower limbs.

For the first strategy, a basic idea is configuring shock-absorbing soles [9], but the shock absorbed by soles is limited because of their limited thickness and damping. In addition, there have been various mechanical devices to address this issue. Jun et al. [10] developed a lower-extremity wearable link mechanism for shock absorption in extreme environments. Jeongsu et al. [11] designed a shock absorption mechanism on shanks for a powered exoskeleton, which can reduce the PGRF effectively for a specific gait. In general, mechanical shock absorption methods always include complex and bulky structures, thus limiting their application in exoskeletons. Moreover, it is difficult to adjust the stiffness and damping parameters of these mechanisms to adapt to different impacts.

For the second strategy, Zelik et al. proposed to reduce the PGRF by trailing ankle push-off just before a contralateral foot strike [12,13], which can reduce the vertical velocity of CoM to reduce potential PGRF. However, the ankle should be powered to provide net-positive work for a preemptive ankle push-off [14], which can not be used for most exoskeletons with passive ankle joints. Moreover, humans can effectively reduce walking impact by knee flexion after strikes [15,16,17]. Further, the stiffness and the damping of human legs are crucial for reducing joint and limb damage on impact [18,19].

In a previous article [20], we proposed a knee trajectory modulation (KTM) for walking-impact reduction in lower-limb exoskeletons. The main idea of the KTM is to imitate the humanoid passive motion of the compliant leg by modulating the active-knee trajectories of the exoskeleton. In this article, the approach is extended. Firstly, we optimized the model parameters of the KTM and called the optimized knee trajectory modulation method OKTM. Secondly, to avoid the modulated knee trajectory leading to an excessive torso pitch angle, we propose a hip trajectory modulation (HTM) for deflection compensation. We performed simulations and experiments for engineering verifications. As a result, modulated trajectories can be used to reduce the PGRF, and the main contributions can be summarized as:

- An optimized knee trajectory modulation (OKTM) is proposed for walking-impact reduction in lower limb exoskeletons.
- An HTM is proposed to compensate for excessive torso pitch deflection caused by the OKTM.
- The proposed approach is validated by simulations and the AIDER lower-exoskeleton system. Results show that the OKTM is effective in reducing the PGRF and that the HTM can reduce torso deflection during walking.

The following sections are organized as follows: Section 2 proposes the implementation of the OKTM and the HTM. Section 3 presents simulations and experimental results on a lower-limb walking-assistance exoskeleton. Finally, we conclude the paper in Section 4 and suggest future work.

## 2. Methods

**Figure 2:**
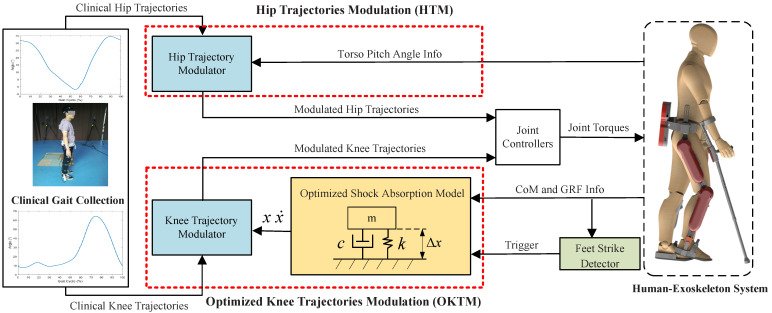
The framework of the proposed approach. The left part is a clinical gait collection by motion-capture systems, e.g., VICON, etc. The right part is a human-exoskeleton system. The proposed approach has two modulations: the OKTM and the HTM. The OKTM is composed of two parts: the OSAM and the knee-trajectory modulator. The OSAM is designed to generate the response of the spring-damping system. The knee-trajectory modulator can integrate clinical gait trajectories with the output of OSAM. In the second part, the HTM can modulate the clinical hip trajectories to stabilize the torso.

This section will introduce the OKTM and the HTM for the impact reduction of lower-limb exoskeletons. Considering that the ankle joints of most exoskeletons are passively driven, we mainly modulate the trajectories of the knee and hip joints. The framework of the proposed approach is shown in Figure 2. Clinical knee and hip trajectories can be acquired via motion capture systems from walking trials. CoM and GRF information can be computed with sensor data sampled from the exoskeleton. The feet strike detector was introduced in a previous paper [20], and it can detect the moments when swing feet fully touch the ground by plantar pressures and trigger the OKTM and HTM. The OKTM comprises two parts: the optimized shock absorption model (OSAM) and the knee-trajectory modulator. The OSAM can deliver an optimized spring-damping system response for hip–ankle distance variation (i.e., knee angle variation). The knee-trajectory modulator can combine the clinical knee trajectories with the output of the OSAM to produce the modulated knee trajectories. In addition, the HTM only includes the hip-trajectory modulator, which can modulate the clinical hip trajectories according to torso pitch-angle information and generate modulated hip trajectories. Finally, the desired reference joint angles and joint torques can be applied in the control of PID high-gain joint controllers.

### 2.1. Optimized Knee-Trajectory Modulation

#### 2.1.1. Optimized Shock-Absorption Model

**Figure 3:**
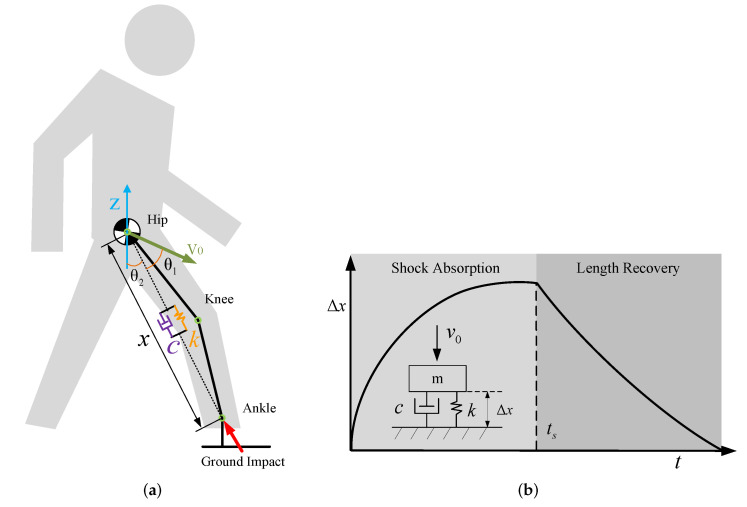
(**a**) To deal with the impact force transmitted from the foot to the CoM, the OSAM was introduced to guide hip–ankle distance variation, where *x* denotes the hip–ankle distance, *k* and *c* are stiffness and damping of the OSAM, respectively, and $V_{0}$ is the velocity of the CoM. (**b**) The typical variation in the hip–ankle distance for shock absorption and length recovery, where Δx denotes the OSAM response, $v_{0}$ is the contact velocity along the leg direction, and $t_{s}$ is the duration of shock absorption.

In this section, we present the OSAM of the OKTM. We focus on the shock absorption in the 2-D sagittal plane. Generally, the CoM of human-exoskeleton systems can be located on hip joints [21]. To deal with the impact force transmitted from feet to the CoM, we considered introducing a spring-damping system as the shock-absorption model, which shows shock-absorption behaviour [11]. In addition, we optimized the parameters of the model, i.e., OSAM. As shown in Figure 3a, the OSAM is placed along the direction from the CoM to the ankle joint, which includes a virtual spring with stiffness *k* and a virtual damper with damping *c*. The hip–ankle distance *x* is planned to track the response of the OSAM for shock absorption after feet strikes, and then restore the original length. The typical OSAM response Δx(t) is shown in Figure 3b, which contains two phases: shock absorption and length recovery. The slight knee-flexion angle for shock absorption is similar to the human walking response to adapt to ground or load [22,23]. The passive ankle joint is not described in detail because it is weakly related to the initial ground-contact impact.

Define t=0 when the swing foot strikes. In the shock absorption duration, the variation in hip–ankle distance Δx(t)=x(0)−x(t) can be expressed in the dynamical equation as:

(1) $$\begin{array}{c} \Delta \ddot{x}+2\xi \omega_{n}\Delta \dot{x}+\omega_{n}^{2}\Delta x=\frac{f_{st}}{m}\\ \xi =\frac{c}{2\sqrt{km}},\;\omega_{n}=\sqrt{\frac{k}{m}} \end{array},$$

where ξ denotes the damping ratio, and $\omega_{n}$ is the natural frequency of the OSAM. In addition, *k* and *c* are stiffness and damping of the OSAM, respectively. The patient and the exoskeleton are physically combined, and the mass of the human-exoskeleton system is *m*. The bodyweight force $f_{st}$ can be computed as:

(2) $$f_{st}=\frac{\mu mg}{cos\;\theta_{2}}-F_{pre},$$

where $\mu =f_{p}/mg$ is the ratio of body weight supported by the landing leg, and $f_{p}$ is the ground vertical-reaction force, which can be obtained from landing-foot pressure sensors in a static step posture. In addition, $\theta_{2}$ is the angle between the support leg and gravity direction, as shown in Figure 3a, and it can be calculated from the inertial measurement unit (IMU) and joint encoder data of the exoskeleton. $F_{pre}$ is the preload force of the spring, which can be used as one parameter to adjust the OSAM response.

As shown in Figure 3b, in the shock absorption duration, the spring and damper can extend the cushioning time and dissipate the impact energy, thus reducing the PGRF. Specifically, the OSAM should be over-damped to avoid vibration when the swing foot strikes; thus, ξ≥1. In addition, the response Δx(t) consists of two parts: response by the applied bodyweight force $\Delta x_{1}\left(t\right)$ and response by the contact velocity $\Delta x_{2}\left(t\right)$.

Response by the applied bodyweight force: The vibration response of over-damped systems can be used, and the OSAM response by the applied bodyweight force ($f_{st}$) can be defined as $\Delta x_{1}\left(t\right)$:

(3) $$\begin{array}{c} \Delta x_{1}\left(t\right)=\frac{f_{st}}{k}\left(1-c_{1}e^{-\left(\xi -\sqrt{\xi^{2}-1}\right)\omega_{n}t}+c_{2}e^{-\left(\xi +\sqrt{\xi^{2}-1}\right)\omega_{n}t}\right)\\ c_{1}=\frac{1}{2\sqrt{\xi^{2}-1}\left(\xi -\sqrt{\left.\xi^{2}-1\right)}\right.}\\ c_{2}=\frac{1}{2\sqrt{\xi^{2}-1}\left(\xi +\sqrt{\left.\xi^{2}-1\right)}\right.} \end{array}.$$

Response by the contact velocity: In addition to the response for gravity, the response caused by inertia should also be considered, i.e., the response by initial ground-contact velocity $\Delta x_{2}\left(t\right)$:

(4) $$\begin{array}{c} \Delta x_{2}\left(t\right)=e^{-\xi \omega_{n}t}\left(c_{3}e^{\sqrt{\xi^{2}-1}\omega_{n}t}+c_{4}e^{-\sqrt{\xi^{2}-1}\omega_{n}t}\right)\\ c_{3}=\frac{v_{0}}{2\omega_{n}\sqrt{\xi^{2}-1}}\\ c_{4}=\frac{-v_{0}}{2\omega_{n}\sqrt{\xi^{2}-1}} \end{array},$$

where the initial ground-contact velocity $v_{0}$ is the component of the CoM velocity $V_{0}$ along the direction of the landing leg, and $v_{0}=V_{0}\;cos\;\theta_{1}$. In addition, $\theta_{1}$ is the angle between the CoM velocity direction and the landing-leg direction, as shown in Figure 3a.

Response of the OSAM: As a linear system, Equations (3) and (4) can be used to calculate the output of the OSAM Δx(t) by the superposition principle:

(5) $$\Delta x\left(t\right)=\Delta x_{1}\left(t\right)+\Delta x_{2}\left(t\right),$$

and the hip–ankle distance planning x(t) can be expressed as:

(6) x(t)=x(0)−Δx(t),

then, we convert the hip–ankle distance planning to knee joint via leg geometry:

(7) $$\theta_{a}\left(t\right)=arccos\;\left(\frac{l_{c}^{2}+l_{t}^{2}-x{\left(t\right)}^{2}}{2l_{c}l_{t}}\right),$$

where $l_{c}$ and $l_{t}$ are calf length and thigh length, respectively.

Parameter optimization of the OSAM: Based on the force analysis of the OSAM, combined with the motion differential Equation (1), the support force of the touchdown leg in the shock absorption process can be expressed as:

(8) $$F=c\Delta \dot{x}+k\Delta x+F_{pre}.$$

As mentioned, the PGRF should be as small as possible in the shock absorption process. Therefore, we formulate this problem as an optimization problem, as follows:

(9) $$\mathrm{argmin}\;F_{max}\left(k,F_{pre}\right)$$

(10) $$\begin{array}{cc} \; & \;s.t.1\;:0<2t_{s}<\omega \%\;T_{n}\\ \; & \;s.t.2\;:0<\Delta \theta_{a}\left(t\right)<\Delta \theta_{\max}\\ \; & \;s.t.3\;:\dot{\Delta x(t)}>0,t\in \left(0,ts\right) \end{array}$$

where $T_{n}$ is the gait period, and ω is an empirical value from human walking to limit the duration of shock absorption and length recovery [22,23]. Thus, end time of the knee-trajectory modulation $2t_{s}$ should be earlier than $\omega \%\;T_{n}$. In addition, the maximum knee-flexion angle in the shock absorption should be limited to $\Delta \theta_{\max}$. Abdel et al. [24] suggested that $\Delta \theta_{\max}$ should not exceed about $22^{\circ }$. Finally, to avoid oscillation, the motion of the shock-absorption process should be unidirectional. There is a nonlinear optimization problem; however, simulated annealing algorithms, BP (back-propagation) algorithms, etc. can be used in the optimization.

#### 2.1.2. Knee-Trajectory Modulator

In this section, we consider a combination of shock-absorbing knee motion $\theta_{a}\left(t\right)$ with the clinically collected knee trajectories θ(t). Firstly, original vibrations of the clinical knee trajectories θ(t) within the shock absorption should be removed, so that motion of the touchdown leg movement can follow the OSAM (Equation (7)) without interference. The vibration-removed knee trajectories $\theta_{b}\left(t\right)$ can be shown as:

(11) $$\theta_{b}\left(t\right)=\left\{\begin{array}{cc} \theta (\omega \%\;T_{n}) & \left(0\leq t<\omega \%\;T_{n}\right)\\ \theta (t) & \left(\omega \%\;T_{n}\leq t<T_{n}\right) \end{array}\right..$$

Note that, to maintain the compressed trajectory’s continuity, we chose the original gait trajectory with θ(0) equal to $\theta (\omega \%T_{n})$.

After shock absorption, the modulated knee trajectory should gradually return to the clinical knee trajectory θ(t). For OSAM, considering that the response of inertia has been dissipated, only the response of gravity is left. A reverse bodyweight force ($-f_{st}$) was input at the end of the shock absorption, and the reverse trajectory was obtained:

(12) $$\theta_{c}\left(t\right)=\left\{\begin{array}{cc} 0 & \left(0\leq t<t_{s}\right)\\ -arccos\;(\frac{l_{c}^{2}+l_{t}^{2}-{\left(x\left(0\right)-\Delta x_{1}\left(t\right)\right)}^{2}}{2l_{c}l_{t}}) & \left(t_{s}\leq t<T_{n}\right) \end{array}\right.,$$

finally, Equation (7), (11), and (12) can be used to calculate the modulated knee trajectory:

(13) $$\theta^{\prime }\left(t\right)=\theta_{a}\left(t\right)+\theta_{b}\left(t\right)+\theta_{c}\left(t\right).$$

**Figure 4:**
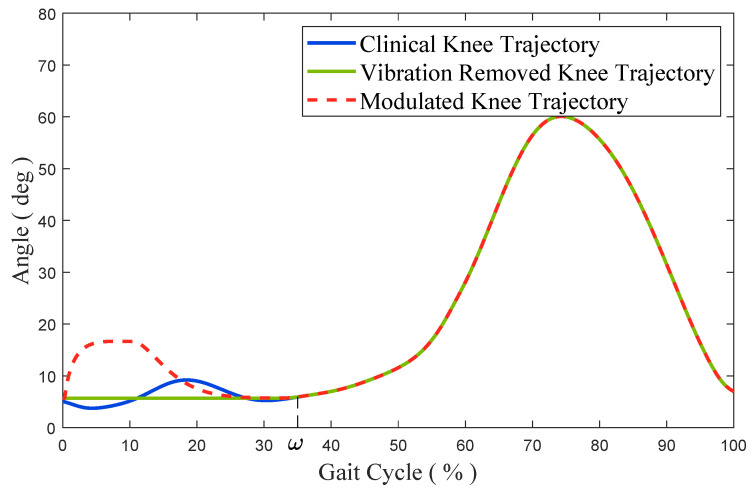
The typical clinical knee-trajectory, vibration-removed knee trajectory, and modulated knee trajectory. After swing-feet strikes, the modulated knee trajectory has a larger amplitude to mimic the OSAM, and returns to the clinical knee trajectory after the shock absorption.

In this way, the modulated knee trajectory $\theta^{\prime }\left(t\right)$ retains the simplicity of the clinical gait and can be used for the shock absorption. As shown in Figure 4, after a foot strike, the modulated knee trajectory has a larger amplitude to mimic the OSAM, and returns to the clinical knee trajectory after the shock absorption.

### 2.2. Hip-Trajectory Modulation

**Figure 5:**
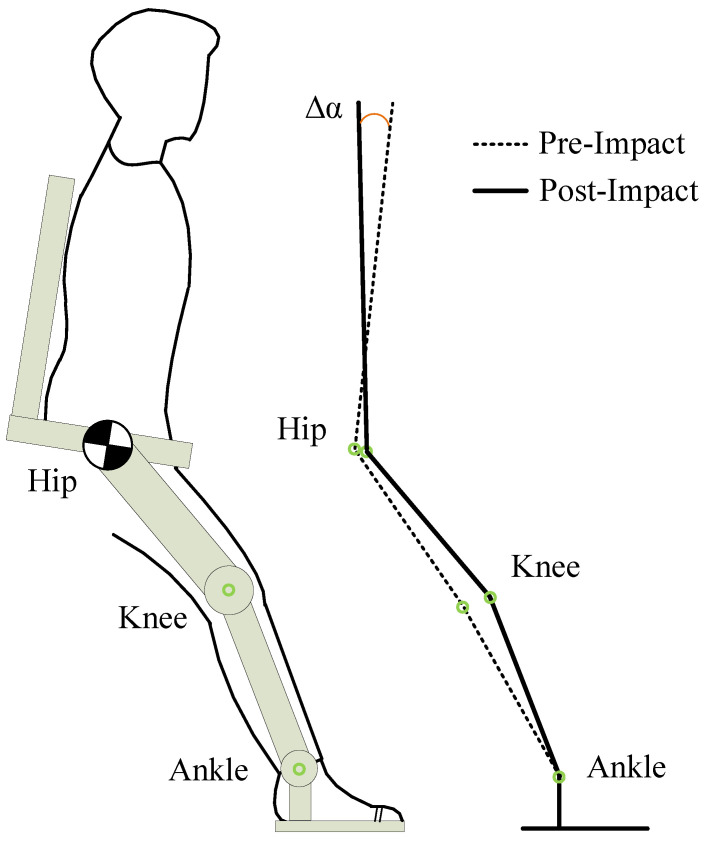
The knee flexion of the OKTM causes an axial rotation in the angle of the torso.

In this section, we introduce the HTM. For human walking, high-amplitude movements of lower limbs are attenuated in the upper body to stabilize the torso and head, especially to maintain the stability of the axial rotation angle of the torso [25]. A knee flexion motion is introduced in the clinical gait by the OKTM. If there is no compensatory mechanism for the hip joint, it will lead to axial rotation of the torso, as shown in Figure 5. Therefore, the HTM is utilized to stabilize the upper body.

Without loss of generality, define the time when the swing foot strikes as 0s. With the IMU attached to the backpack of the exoskeleton, the actual torso angle α(t) during shock absorption can be measured, and we can establish the following HTM:

(14) $$\beta^{\prime }\left(t\right)=\left\{\begin{array}{cc} \beta (t)+\Delta \alpha & \left(0\leq t<\omega \%T_{n}\right)\\ \beta (t) & \left(\omega \%T_{n}\leq t<T_{n}\right) \end{array}\right.,$$

where $\beta^{\prime }\left(t\right)$ is the modulated hip trajectory, and the clinical hip trajectory is expressed as β(t). The torso deviation angle can be computed as $\Delta \alpha =\hat{\alpha }-\alpha \left(t\right)$, among which $\hat{\alpha }$ is the measured torso angle in clinical trials. More specifically, the $\hat{\alpha }$ remains almost constant during support phases.

**Figure 6:**
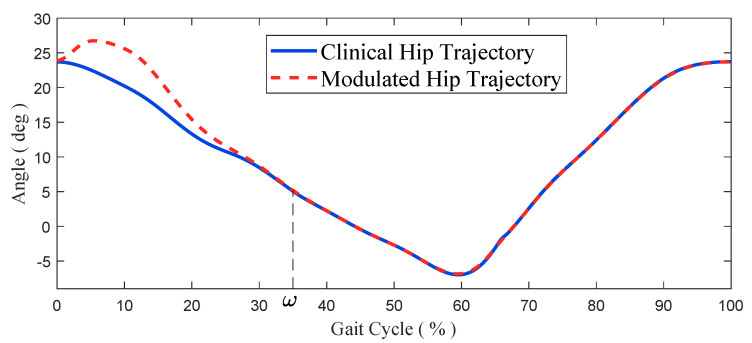
The typical clinical hip trajectory and modulated hip trajectory. Stabilization of the torso by compensation of the hip trajectory after feet strikes.

The clinically collected hip trajectory and the modulated hip trajectory are shown in Figure 6. The modulated hip trajectory lagged in extension to compensate for the torso tilt caused by the OKTM and return to the clinical trajectory after the shock absorption.

## 3. Experiments and Discussion

In this section, we present the simulation results of the OKTM and the experimental walking results of the AIDER exoskeleton.

### 3.1. Simulation of the OKTM

We used numerical simulation to validate the performance of the OKTM. In simulations, we mainly focused on the performance of OSAM in the shock absorption process, so the process of length recovery after shock absorption was not demonstrated. The leg support forces and knee-angle variations were simulated for two typical initial contact velocities. The simulation environment was implemented in MATLAB using a sampling time of 0.1 ms. The basic parameters of the simulation were set as shown in Table 1.

**Figure 7:**
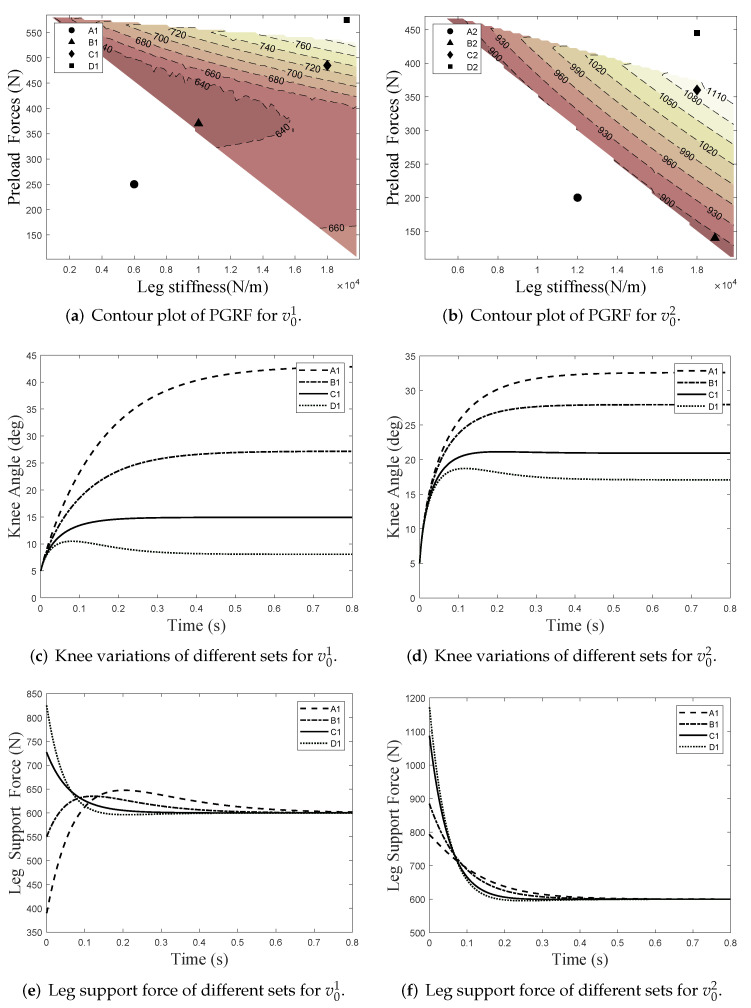
Simulation results for $v_{0}^{1}$ and $v_{0}^{2}$ with different sets.

Contour maps of computational PGRF for $v_{0}^{1}$ and $v_{0}^{2}$ are proposed in Figure 7a,b. In each contour map, we selected four typical points from A to D, i.e., four sets of leg stiffness *k* and preload-forces $F_{pre}$ for detailed display. On the one hand, the knee angles of the landing leg are simulated in Figure 7c,d, respectively. On the other hand, the support force of the landing leg are simulated in Figure 7e,f, respectively. Finally, the numerical results are shown in Table 2 and Table 3.

In Figure 7a,b, the colored parts of contour plots are feasible domains for the OSAM, i.e., satisfying Equation (10). Although set-A (A1 and A2, with lower $F_{pre}$ and *k*) contributes to the reduction in the PGRF, it leads to excessive knee flexion angles, (as shown in Figure 7c,d, which may cause the opposite foot to scuff the ground during swings. According to the experience of clinical trials, the variation in knee flexion during shock absorption should not exceed about $22^{\circ }$ [24].

The simulation results for set-C (C1 and C2, with higher $F_{pre}$ and *k*) and set-D (D1 and D2, with higher $F_{pre}$ and *k*) are as expected, with excessive stiffness *k* and preload $F_{pre}$ both increasing the leg support force *F* at the initial moment. In addition, an excessive preload makes the gravity response $\Delta x_{1}\left(t\right)$ amplitude more minor than the velocity response $\Delta x_{2}\left(t\right)$ amplitude, so the total response Δx(t) will fall back after reaching the maximum value, making the knee motion oscillate, as shown in the D1 curves in Figure 7c,d.

Set-B(B1 and B2, with optimized $F_{pre}$ and *k*) have maximum knee flexion angles in the stability domains, and can effectively reduce the PGRF. It is notable that, when $F_{pre}+cv_{0}>\mu mg/cos\theta_{2}$, the leg support force *F* is monotonically decreasing, i.e., it reaches the maximum at the initial moment, e.g., C1 and D1 in Figure 7e. However, when $F_{pre}+cv_{0}<\mu mg/cos\theta_{2}$, the leg support force *F* will gradually increase to the maximum with compression of the leg length, and then, as the speed decreases, the damping force decreases, making the support force gradually decrease again to $\mu mg/cos\theta_{2}$, e.g., A1 and B1 in Figure 7e. In general, the optimizer of the OSAM selected for set-B has better performance when the constraints are satisfied.

### 3.2. Experiments on the AIDER Exoskeleton System

**Figure 8:**
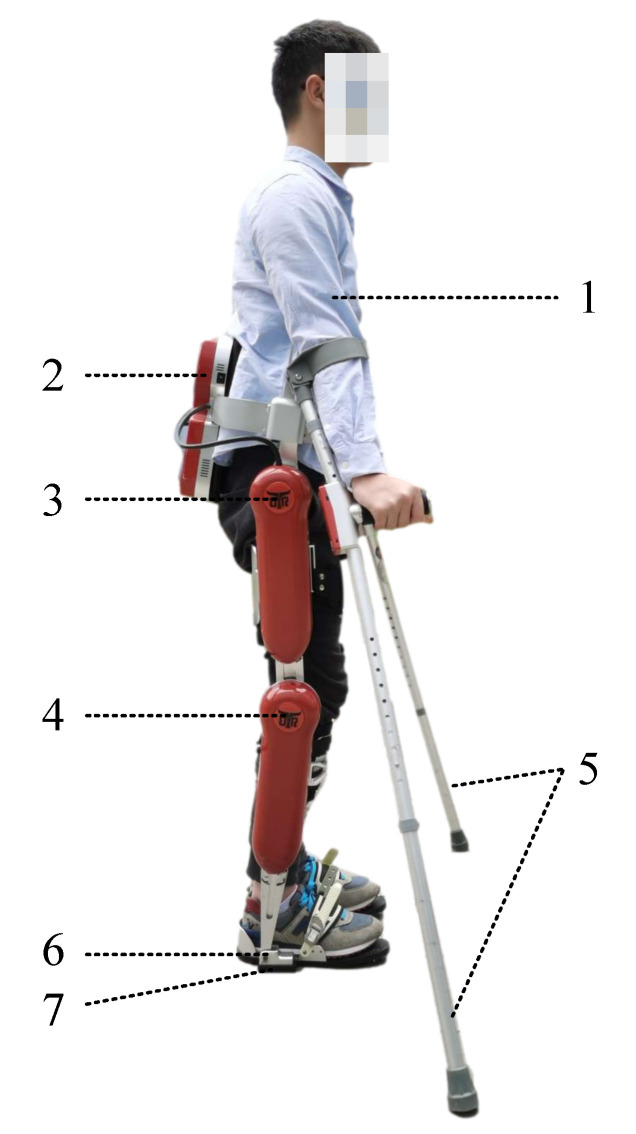
The AIDER exoskeleton system: 1: pilot. 2: backpack, with an embedded computer. 3 and 4: hip and knee flexion/extension joint activated by dc servo motors. 5: crutches. 6: passive ankle flexion/extension joint. 7: shoes with plantar pressure sensors inside.

Description of the AIDER: The AIDER is an exoskeleton robot designed for paraplegic patients, developed jointly by our lab in the university and Buffalo Robotics Tech Co. With the assistance of the AIDER, patients can cross common scenarios such as flat ground, stairs, and ramps. As shown in Figure 8, patients’ thighs, calves, torso, and feet can be separately bound to exoskeleton segments by ties, enabling patients’ movements to follow the exoskeleton. The AIDER’s hip flexion/extension joints and knee flexion/extension joints are driven actively by servo motors (Maxon EC 40, 170 W), while the ankle joints are passive joints embedded with low-stiffness elastic elements. AIDER is equipped with three types of sensors: IMU, encoder, and pressure sensor. The IMU in the backpack of the AIDER provides real-time feedback on the position and posture of the torso. Absolute-value encoders and incremental encoders in joints (hips, knees, and ankles) provide real-time feedback on these joints’ angles and angular velocities. The pressure sensors distributed on the bottom of the feet provide feedback on the pressure values for triggering the trajectory modulations. The AIDER uses a distributed control system, where individual sensors and drives act as nodes communicating with the main controller via the bus. Finally, patients can operate two separate buttons on the crutches to access different state machines.

Experiment setup: We captured some joint trajectories from healthy subjects in walking experiments using the motion capture system Vicon, and these trajectories were trimmed to maintain smoothness and cycle continuity. A spinal-cord-injured pilot (AIS A, with a complete loss of motor function [26]) tested the proposed method on the AIDER. Before that, he had more than one year of experience in using the AIDER.

In experiments, patients can trigger the exoskeleton’s stride by clicking buttons on the crutches. In addition, the crutches can be used to maintain balance in the sagittal plane. The touchdown-leg bodyweight supported rate μ was measured in a static step posture. The experimental data, such as GRF, torso pitch angle, and joint angles, were recorded in the embedded computer. Parametric values of the experiment are given in Table 4.

**Figure 9:**
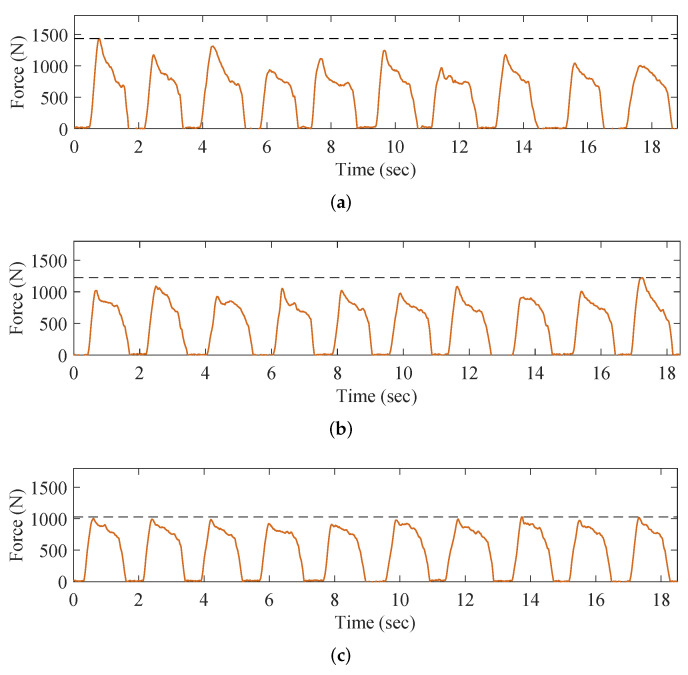
GRF data obtained from shoes sensors when walking at about 0.5 m/s. (**a**) The GRF data when walking with CG. (**b**) The GRF data when walking with MGA. (**c**) The GRF data when walking with MGB.

**Figure 10:**
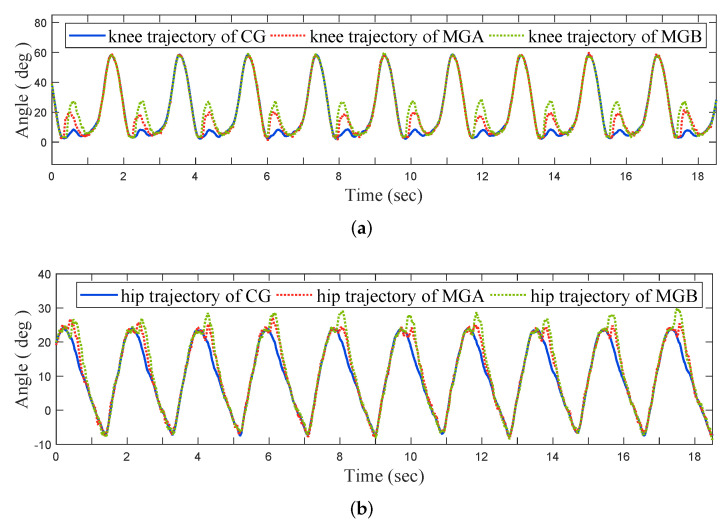
Joint angles of the AIDER walking at about 0.5 m/s. (**a**) Knee joint trajectories. (**b**) Hip joint trajectories.

Experimental Results of the OKTM: To verify the effectiveness of the OKTM, we experimented with three gaits on the AIDER: The Clinical Gait (CG); Modulated Gait B (MGB), where parameters are optimized; and Modulated Gait A (MGA), where parameters are suboptimal (with $k=0.9\;k_{max}$ and $F_{pre}=0.9\;F_{pre\;max}$, where $k_{max}$ and $F_{pre\;max}$ are maximum values of *k* and $F_{pre}$ satisfies the constraints in Equation (10), respectively). In addition, we tried two different walking speeds with these gaits: about 0.5 m/s and 0.25 m/s. In Figure 9, the GRF for three different gaits when walking at about 0.5 m/s are presented. The joint angles corresponding to Figure 9 are shown in Figure 10. For comparison, we aligned the gait period of MGA and MGB to that of CG.

The performance of the OKTM was evaluated by comparing the PGRF between the three gaits. Periodic occurrence of force duration can be interpreted as the standing phases, e.g., 0.5 s to 1.7 s in Figure 9a. As expected, the PGRF often appears at the beginning of foot–ground collisions, e.g., 0.7 s in Figure 9a. This agrees with the result obtained by Jeongsu et al. [11]. At moments of strikes, movements of landing legs follow predefined trajectories and behave rigidly, resulting in sudden CoM velocity variations, thus leading to excessive PGRF. In contrast, the flexible movement of knee joints can reduce the PGRF, as shown in Figure 9b,c. In addition, the optimized knee stiffness and preload allow for more extended and more angular shock absorption, resulting in a lower PGRF for the MGB than the MGA. Table 5 and Table 6 compare two walking speeds by comparing the mean values and standard deviation values. The results suggest that the PGRF after strikes was reduced by implementing the proposed OKTM method. Periodic micro-force duration can be analyzed as swing phases, e.g., 1.6 s to 2.2 s in Figure 9c. Lower GRF during swing phases in Figure 9b,c indicate that swing feet are not scraping ground.

The knee and hip trajectories of the MGA and MGB are shown in Figure 10a,b, respectively. After feet strikes, both knee and hip joints exhibit flexion movements in the MGA and MGB. Since the MGB was set with a lower stiffness and preload than MGA, the shock absorption angle of the MGB tended to be greater than that of MGA, but none exceeded the boundary value in Equation (10), as shown in Table 5 and Table 6.

**Figure 11:**
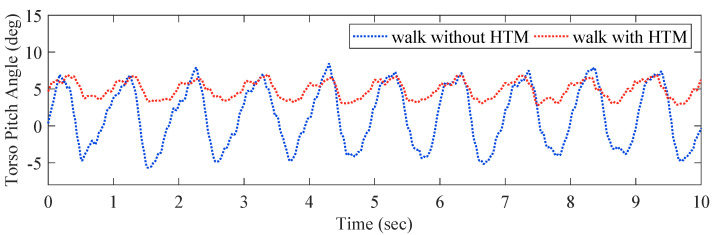
The torso pitch angles when walking with the OKTM.

*Experimental Results of the HTM:* To verify the effectiveness of HTM, we show the effect of the HTM on the torso pitch angle when walking with the OKTM, as shown in Figure 11 and Table 7. In human walking, to easily shift weight to the front support leg, the pitch angle of the torso reaches a maximum before each foot–ground collision, and the torso is slightly tilted forward at this moment. When walking without the HTM, the OKTM modulates the knee joint of the front leg to flex rapidly, causing the torso pitch angle to decrease rapidly, and the torso is slightly tilted backward. This increases the burden on the patient’s sagittal balance and defeats the original purpose of keeping the torso angle stable during walking. In contrast, the introduction of the HTM can effectively reduce torso sway after swing-foot strikes, helping patients to walk more stably.

*Limitations of the proposed approach*: Firstly, we recruited only one patient to validate the proposed method, and the validity and stability of the proposed method need to be tested with a large number of patients. Secondly, more accurate human-exoskeleton models, such as low stiffness passive ankle joints, individual patient differences, etc., should be considered. Finally, considering the AIDER exoskeleton is a biomedical device, we have only conducted engineering validations, and further clinical studies are yet to be conducted. Thus, some areas still need to be addressed in the future.

## 4. Conclusions and Future Works

In this work, we proposed an approach to reduce walking impact by the OKTM for lower limb exoskeletons. In addition, the HTM was proposed to compensate for torso pitch angle deviation due to the OKTM. In the OKTM, we mapped the motion of the spring-damped system to exoskeleton knee joints, and optimized parameters of the OKTM, as a way to reduce the PGRF of human-exoskeleton systems. In the HTM, we compensate for hip trajectory during shock absorption via IMU data. Finally, the effectiveness of the method was verified by simulations and experiments. Simulation results show that the OKTM can not only effectively reduce the PGRF, but also take into account the monotonicity and maximum value of the modulated trajectories. Experimental results further validate the effectiveness of the OKTM, and that HTM can assist in reducing the torso pitch angle deflection caused by the OKTM. In the future, we will explore the extension of the method to the sagittal plane, and conduct further clinical trials.

## Figures and Tables

**Table 1:** Simulattion settings.

Description	Value
CoM mass m	80 kg
Gravitational acceleration *g*	9.8 m/s${}^{2}$
Calf length $l_{c}$	0.44 m
Thigh length $l_{t}$	0.41 m
Initial ground contact knee angle θ(0)	5 deg
Initial ground contact velocities $v_{0}^{1}$	0.1 m/s
Initial ground contact velocities $v_{0}^{2}$	0.3 m/s
Damping ratio of OSAM ξ	1.05
Landing leg bodyweight supported rate μ	0.7
The angle $\theta_{2}$	$20^{\circ }$

**Table 2:** Simulation numerical results of $v_{0}^{1}$.

Initial Ground Contact Velocities $v_{0}^{1}=0.1$ m/s					
	k **[N/m]**	$F_{\mathrm{pre}}$ **[N]**	$\Delta \theta_{\max}$ **[deg]**	PGRF **[N]**	**Description**
A1	6000	250	37.4	647.6	Excessive Δθ
B1	10,000	370	21.9	635	Optimal set
C1	18,000	485	9.8	727.4	Inferior PGRF
D1	19,200	575	5.5	825.4	Inferior PGRF & leg vibrations

**Table 3:** Simulation numerical results of $v_{0}^{1}$.

Initial Ground Contact Velocities $v_{0}^{1}=0.1$ m/s					
	k **[N/m]**	$F_{\mathrm{pre}}$ **[N]**	$\Delta \theta_{\max}$ **[deg]**	PGRF **[N]**	**Description**
A1	12000	200	28.4	793.8	Excessive Δθ
B1	18,900	140	22	885.2	Optimal set
C1	18,000	360	15.9	1087	Inferior PGRF
D1	18,000	445	13.7	1172	Inferior PGRF & leg vibrations

**Table 4:** Experiment setings.

Description	Value
Pilot weight $m_{h}$	60 kg
Exoskeleton weight $m_{r}$	25 kg
Damping ratio of SAM ξ	1.05
Landing-leg bodyweight supported rate μ	0.75

**Table 5:** Walking PGRF at about 0.5 m/s.

Walking Gaits	*k*[N/m] (Mean)	Fpre [N] (Mean)	max$\Delta \theta_{a}$[deg] (Mean)	PGRF [N] (Mean ± SD)	Decline
CG	/	/	/	1140 ± 150	/
MGA	19140	350	15.4	1029 ± 87	10.70%
MGB	19100	130	22	930 ± 45	22.50%

**Table 6:** Walking PGRF at about 0.25 m/s.

Walking Gaits	*k*[N/m] (Mean)	Fpre [N] (Mean)	max$\Delta \theta_{a}$[deg] (Mean)	PGRF [N] (Mean ± SD)	Decline
CG	/	/	/	1030 ± 138.5	/
MGA	18281	504.3	15.6	952 ± 71	8.10%
MGB	14452	332.5	22	893 ± 60	15.30%

**Table 7:** The torso pitch angles.

	Maximum Torso Pitch Angle [deg]	Minimum Torso Pitch Angle [deg]
	(Mean ± SD)	(Mean ± SD)
Walk without HTM	6.53 ± 0.45	−4.11 ± 0.51
Walk with HTM	6.22 ± 0.3	2.72 ± 0.25

## Data Availability

The original data contributions presented in the study are included in the article; further inquiries can be directed to the corresponding authors.
